# Intrinsic Disorder in the Human Spliceosomal Proteome

**DOI:** 10.1371/journal.pcbi.1002641

**Published:** 2012-08-09

**Authors:** Iga Korneta, Janusz M. Bujnicki

**Affiliations:** 1 Laboratory of Bioinformatics and Protein Engineering, International Institute of Molecular and Cell Biology in Warsaw, Warsaw, Poland; 2 Bioinformatics Laboratory, Institute of Molecular Biology and Biotechnology, Faculty of Biology, Adam Mickiewicz University, Poznan, Poland; University of California San Diego, United States of America

## Abstract

The spliceosome is a molecular machine that performs the excision of introns from eukaryotic pre-mRNAs. This macromolecular complex comprises in human cells five RNAs and over one hundred proteins. In recent years, many spliceosomal proteins have been found to exhibit intrinsic disorder, that is to lack stable native three-dimensional structure in solution. Building on the previous body of proteomic, structural and functional data, we have carried out a systematic bioinformatics analysis of intrinsic disorder in the proteome of the human spliceosome. We discovered that almost a half of the combined sequence of proteins abundant in the spliceosome is predicted to be intrinsically disordered, at least when the individual proteins are considered in isolation. The distribution of intrinsic order and disorder throughout the spliceosome is uneven, and is related to the various functions performed by the intrinsic disorder of the spliceosomal proteins in the complex. In particular, proteins involved in the secondary functions of the spliceosome, such as mRNA recognition, intron/exon definition and spliceosomal assembly and dynamics, are more disordered than proteins directly involved in assisting splicing catalysis. Conserved disordered regions in spliceosomal proteins are evolutionarily younger and less widespread than ordered domains of essential spliceosomal proteins at the core of the spliceosome, suggesting that disordered regions were added to a preexistent ordered functional core. Finally, the spliceosomal proteome contains a much higher amount of intrinsic disorder predicted to lack secondary structure than the proteome of the ribosome, another large RNP machine. This result agrees with the currently recognized different functions of proteins in these two complexes.

## Author Summary

In eukaryotic cells, introns are spliced out of proteincoding mRNAs by a highly dynamic and extraordinarily plastic molecular machine called the spliceosome. In recent years, multiple regions of intrinsic structural disorder were found in spliceosomal proteins. Intrinsically disordered regions lack stable native three-dimensional structure in solutions, which makes them structurally flexible and/or able to switch between different conformations. Hence, intrinsically disordered regions are the ideal candidate responsible for the spliceosome's plasticity. Intrinsically disordered regions are also frequently the sites of post-translational modifications, which were also proven to be important in spliceosome dynamics. In this article, we describe the results of a structural bioinformatics analysis focused on intrinsic disorder in the spliceosomal proteome. We systematically analyzed all known human spliceosomal proteins with regards to the presence and type of intrinsic disorder. Almost a half of the combined sequence of these spliceosomal proteins is predicted to be intrinsically disordered, and the type of intrinsic disorder in a protein varies with its function and its location in the spliceosome. The parts of the spliceosome that act earlier in the process are more disordered, which corresponds to their role in establishing a network of interactions, while the parts that act later are more ordered.

## Introduction

In eukaryotic cells and certain viruses that infect them, the coding sequences (exons) of most protein-coding genes are interrupted by noncoding regions (introns). Following the transcription of an entire gene into a precursor messenger RNA (pre-mRNA), the introns are excised and the exons are spliced together to form a functional mRNA. The splicing reaction is catalyzed by a large macromolecular ribonucleoprotein (RNP) machine termed the spliceosome. The most common form of the spliceosome is composed primarily of five small nuclear RNA (snRNA) molecules: U1, U2, U4, U5 and U6, and 45 proteins, arranged into snRNP particles. Seven mutually related Sm proteins are common to all spliceosomal snRNP apart from the U6, which contains a set of related “like-Sm” (Lsm) proteins [1]. The Sm or Lsm proteins form a ring structure that acts as a platform to support the snRNA [2]. Apart from Sm and Lsm heptamers, all other proteins in the human snRNP subunits are unique (review: [3]).

Apart from the snRNP proteins, approximately 80 proteins are abundant in the human spliceosome and reported to be essential to the process of spliceosome-dependent splicing [4], while results of proteomics analyses [4]–[7] yield up to over 200 proteins *in toto*. Non-snRNP splicing factors are divided into independent protein splicing factors and proteins that combine into multiprotein complexes auxiliary to the spliceosome: the hPrp19/CDC5L (NTC) complex, the exon-junction complex (EJC), the cap-binding complex (CBP), the retention-and-splicing complex (RES), and the transcription-export complex (TREX). Spliceosomal proteins are richly phosphorylated, as well as undergo other types of post-translational modifications (review: [8]).

A rare class of introns exists (<1% of all introns in human) that are excised by the so-called minor spliceosome [9]. This low-abundance spliceosome variant contains a U5 snRNP identical to the one from the major spliceosome and four snRNPs with snRNAs U11, U12, U4atac, and U6atac snRNAs that are distinct from, but structurally and functionally analogous to, U1, U2, U4, and U6 snRNAs, respectively. Some proteins specific to the minor spliceosome have been found [10].

The primary activity of the spliceosome, i.e. the excision of introns and ligation of exons, requires the correct working of several additional functionalities of the spliceosomal machinery: recognition of the 5′ and 3′ splice sites (intron/exon definition), mutual recognition of spliceosome subunits and correct spliceosome assembly, spliceosome remodeling and regulation (review: [11]). In the course of the splicing reaction, the snRNP subunits combine and detach from one another and from the pre-mRNA, forming in turn the so-called E (entry), A, B, B* (B-activated), and C complexes. For the major spliceosome, the U1 and the U2 snRNPs perform the initial scanning of the pre-mRNA for intron sites, while the actual two-step splicing reaction occurs after the addition of a U4/U6.U5 tri-snRNP entity and the elimination of the U1 and U4 snRNPs from the complex, at the assembled interface of the pre-mRNA substrate and U2, U5, and U6 snRNAs (complex C). For the minor spliceosome, the U11/U12 di-snRNP performs the role of the U1 and U2 snRNPs, while the U4atac/U6atac di-snRNP performs the role of the U4/U6 di-snRNP (review: [12]). The early recognition and assembly of the splicing reaction (E/A complex formation) rely on the use of multiple weak binary interactions to ensure flexibility. On the other hand, later stages of the splicing reaction (B, B-act, C complexes) involve enzymatic catalysis [11]. Each of the stages of the splicing reaction has its own set of associated non-snRNP proteins [4].

Splicing has been associated with intrinsic protein disorder [13]. Intrinsically disordered regions (IDRs) lack stable, well-defined three-dimensional structure (review: [14]). IDRs frequently contain low-complexity regions and repeats, although they may also contain conserved linear motifs embedded in the less conserved regions (ELMs; [15]). IDRs are not necessarily completely unfolded. In particular, some IDRs may contain stable preformed secondary structure elements in isolation [16], while others may switch from disorder to order (i.e. exhibit “dual personality”) depending on the environment, for instance upon binding to other proteins [17], [18].

As they lack tertiary structure under many or all conditions, IDRs are more flexible and plastic than the rigid structures of globular domains. Disorder may increase the speed of intermolecular binding and unbinding and make interactions weaker [14]. As a result of these properties, IDRs are found in a variety of molecular functions, which include forming linkers between structured domains, being sites of post-translational modifications, and sites of protein-protein and protein-RNA recognition [19]. The large interaction capacity of IDRs predisposes them to organizing the assembly of complexes; disorder is a characteristic feature of “hub” proteins that interact with many partners, and, notably for spliceosome research, disordered proteins are common in large complexes [20]. Among RNP complexes, the ribosome in particular illustrates an RNA-related structural function for disordered proteins. Many ribosomal proteins contain long disordered extensions attached to ordered globular bodies [21] that, upon the formation of the ribosome complex, become ordered and penetrate into the macromolecule core formed by the rRNA [22], [23]. In other words, the long disordered extensions become the “mortar” of the macromolecule that fills in gaps in the rRNA and stabilizes it.

The subject of intrinsic disorder of the spliceosome has not yet been systematically analyzed for the entirety of the spliceosomal proteome. As an essential step towards broadening our understanding of the functioning of the spliceosome, we have carried out a bioinformatics analysis of intrinsic disorder within the human spliceosomal proteome. We discovered that almost half of the residues within the human spliceosomal proteins are disordered, and that the distribution of intrinsic disorder is uneven across the spliceosome. The spliceosome is divided into three layers: a rigid inner core that performs the precise operations required to effect splicing catalysis, a middle layer of disorder that acquires structure in spliceosome-bound proteins, and a fluid outer layer of disordered regions that do not acquire structure and that are responsible for the establishment of a matrix of weak interactions in the initial stages of the splicing process.

## Results/Discussion

### The human spliceosome is highly disordered

Initially, we predicted the average intrinsic disorder content of 122 core proteins of the major human spliceosome, including all abundant proteins *sensu* Agafonov et al. [4] (Table S1). This prediction was carried out in two stages. The initial fully automated analysis, carried out via the GeneSilico MetaDisorder server [24], estimated the intrinsic protein disorder content in the 122 human spliceosomal proteins at 53.5%, and at 45.2% for 45 proteins of the snRNP subunits of the major spliceosome (each Sm protein counted once). Subsequently, we adjusted manually the predictions of order/disorder boundaries of IDRs based on structural predictions yielded by the GeneSilico MetaServer [25]. This manual correction shifted the disorder estimate downwards in some cases by as much as 10%, to an intrinsic disorder content estimate of 44.0% for all the 122 proteins of the major spliceosome, and 34.1% for the snRNP proteins. Nevertheless, even after the correction, at least 98 out of the 122 core spliceosomal proteins (80.3%) were predicted to contain at least one IDR≥30 residues.

An intrinsic disorder content estimate of 44.0% is twice the average value for all human proteins as calculated on the basis of genome-based predictions, which is 21.6% [26]. The predicted fraction of 80.3% of proteins with at least one IDR≥30 residues contrasts against the calculated fraction of 35.2% for the entire human proteome [26]. Although different methods of prediction of intrinsic disorder content differ in their estimates, altogether the human spliceosomal proteome contains a high amount of intrinsic disorder. This finding will have a significant impact on further studies involving spliceosomal proteins.

### Early human spliceosomal proteins are more disordered than late proteins

To determine whether there was any variation of disorder content throughout the complexes forming the spliceosome at different stages of the splicing reaction, we analyzed the fraction of predicted intrinsic disorder for different groups of proteins of the spliceosome complex. For this analysis, we divided the spliceosome proteins in our dataset into several groups based on proteomics data as well as included eight proteins of the U11/U12 di-snRNP of the minor spliceosome (Table S1). As most of the U11/U12 proteins are structurally and functionally related to proteins of the U1 and U2 snRNPs [10], we expected that they would have a similar IDR content to the U1 and U2 snRNP subunit proteins.

**Figure 1:**
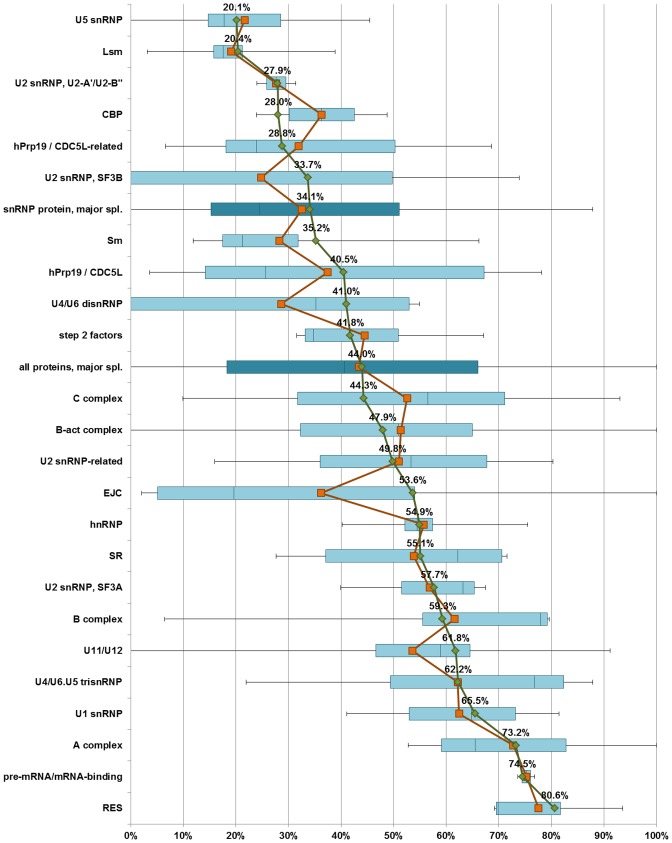
Intrinsic disorder content of the various groups of core spliceosome proteins. In deeper shades are marked the values for all proteins of the snRNP subunits of the major spliceosome (“snRNP proteins, major spl.”) and for all the proteins of the major spliceosome (“all proteins, major spl.”). The orange line indicates means calculated per-protein (disorder fraction was calculated for each protein first, and then a mean was taken out of this) while the green line indicates means calculated per-residue (the number of all disordered residues in a protein group divided by the total length of proteins in the group). Per-residue means are indicated above the line. Spliceosome protein groups are ordered according to per-residue means.

Different groups of spliceosome proteins differ in their predicted disorder content (Figure 1). In particular, proteins of the U1 snRNP, U2 SF3A, U11/U12 di-snRNP, U2-related and U4/U6.U5 tri-snRNP-specific proteins are predicted to be more disordered than average spliceosome proteins (>44.0% disorder content). Of these groups of proteins, all apart from the U4/U6.U5 tri-snRNP-specific proteins are “early” proteins associated with the early stages of splicing. On the other hand, U2 SF3B, U4/U6 di-snRNP, U5 snRNP, Sm and Lsm proteins are predicted to be more ordered than average (<44.0% disorder content). The Sm and Lsm proteins comprise scaffolds for snRNA, and especially proteins of the U4/U6 di-snRNP and U5 snRNP may be responsible for assisting in splicing catalysis. Among auxiliary protein complexes, the retention-and-splicing (RES) complex, whose function is the retention of unspliced pre-mRNAs in the nucleus [27], is predicted to be extremely disordered (80.6%), while the cap-binding complex (CBC) is more ordered than average (28.0%). Two other complexes, hPrp19/CDC5L and EJC, both of which have multiple functions, situate in between (40.5% and 53.6% disorder content, respectively). Finally, while all the groups of transiently binding non-snRNP spliceosomal proteins are predicted to be more disordered than average for all spliceosomal proteins, the early A-complex proteins are predicted to be the most disordered in this group, followed by B-complex proteins, B-act complex proteins, and C-complex proteins.

### Early human spliceosomal proteins contain more compositionally biased disorder than late proteins

As no external standardized annotation scheme was available for IDRs in the spliceosomal proteins, we developed a classification based on their predicted primary and secondary structure features. We divided the spliceosomal IDRs into three classes: regions with consistently predicted secondary structure (SS) elements (henceforth “disorder with SS” or “IDR with SS”), long (≥25 residues) compositionally biased IDRs without predicted secondary structure elements (henceforth “compositionally biased disorder/IDR”), and other IDRs, which we omitted from further analyses (Figure S1). Several types of compositionally biased regions without predicted SS elements that frequently appear throughout the spliceosomal proteome had been previously described in literature. For these compositionally biased IDR types, we sought to define relevant standard IDR subclasses within our classification (RS-like, poly-P/Q, G-rich; see Methods for details).

**Figure 2:**
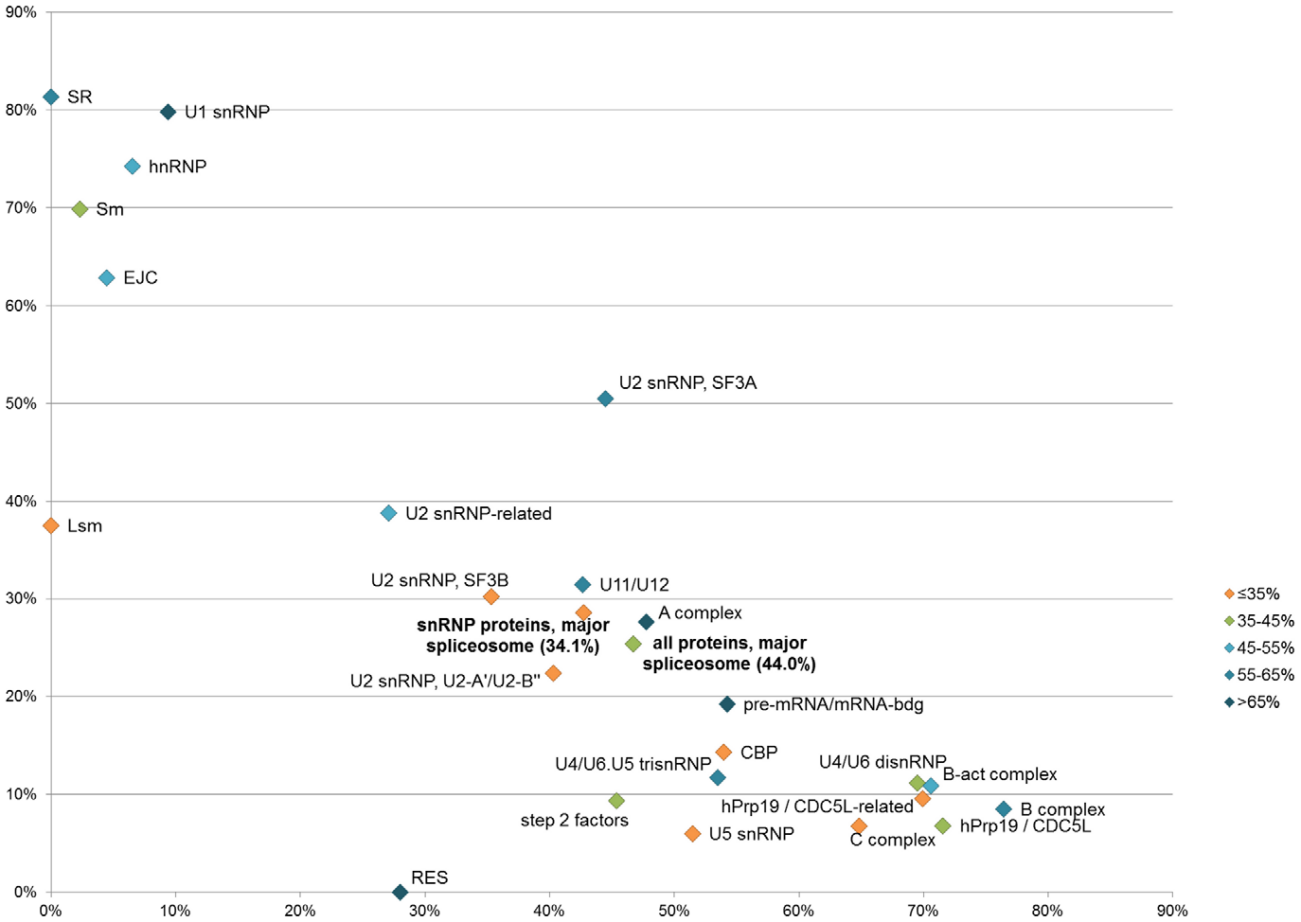
Types of disorder in core spliceosomal proteins. Compositionally biased disorder (Y-axis) vs. disorder with SS (X-axis). Datapoints are colored according to predicted total per-residue disorder content. Groups of all proteins of the major spliceosome and all proteins of the snRNP subunits of the major spliceosome are indicated in bold.

Having annotated the IDRs, we analyzed the distribution of different types of disorder across different groups of human spliceosome proteins. Different groups of spliceosome proteins are predicted to differ in the type of disorder they contain (Figure 2, Figure S2). The heptameric complexes of Sm and Lsm proteins are predicted to contain mainly compositionally biased disorder without secondary structure elements (69.9% of all disorder). Correspondingly, crystal structures of the Sm complex lack most of the predicted disordered regions (example PDB ID: 2Y9A, [28]) and show a stable ungapped platform, which suggests that disorder in Sm and Lsm proteins is located outside of the ordered torus. Protein groups that are present earlier in the course of the splicing process and that are in general highly disordered (U1, U2 SF3A, U11/U12, U2-related, SR, hnRNP, A-complex proteins) are predicted to contain more disorder with predicted compositional bias and less disorder with SS than late proteins. Similarly to 2Y9A, the majority of predicted disorder of the U1 snRNP-specific proteins included in the crystal structure of the U1 snRNP (PDB ID: 3CW1; [29]) is missing from the crystal structure. Also similarly to 2Y9A, almost all compositionally biased disorder is missing from the structure, while almost all predicted disorder with SS is present. Notably, also the EJC, whose post-splicing functions in exon ligation and mRNA transport involve mRNA binding, also exhibits a high content of compositionally biased disorder (62.9%). The RES complex also contains long regions of disorder with very little predicted secondary structure, but we could not unambiguously divide these regions into subregions with different compositional bias.

Among different types of compositionally biased disorder, RS-like IDRs are found in all groups of early proteins, while poly-P/Q and miscellaneous noncharged IDRs are predicted to be concentrated mainly in the U1, U2, U11/U12 and U2-related proteins. Domain-length (≥100 residues) hnRNP-type G-rich regions are found only in hnRNP proteins, but short (<100 residues) hnRNP-like G-rich regions are found, in addition to SR and Sm proteins, in A-complex and U2-related proteins (Table S2). Based on the widespread distribution of compositionally biased IDRs in spliceosomal proteins, we speculate that interactions mediated by these IDRs may be in fact more common and important than suggested by the particular cases studied before. In particular, the role of glycine-rich regions in many spliceosomal proteins is unknown and requires further study. Based on the fact that RS-like and glycine-rich disordered regions frequently appear in the same proteins (e.g. SF2/ASF, TRAP150) and in proteins that interact with each other and/or interact with the same RNA (SR, hnRNP), we also suggest that these two types of regions may interact with each other directly. If so, also RS-like and glycine-rich regions from other proteins may interact with one another. This interaction may be important for the regulation of splicing and definition of intron/exon boundaries, and, by extension, for the regulation of alternative splicing.

In contrast to early proteins, proteins of the later stages of splicing are often predicted to contain high amounts of disorder with SS. These proteins include proteins of the U5 snRNP and U4/U6 di-snRNP, proteins specific to the U4/U6.U5 tri-snRNP entity, hPrp19/CDC5L, step 2 catalytic factors, as well as B, B-act and C-complex proteins. Most of these protein groups are also predicted to be relatively ordered. In particular, for the isolated proteins of the U5 snRNP, which is predicted to be the least disordered of all the snRNP subunits, over a half of the disordered residues are predicted to be in IDRs with SS. We suggest that, in the case of proteins of larger complexes, disorder with SS may acquire structure as the individual proteins of the complex come together. If so, the U5 snRNP may be almost completely ordered when the proteins come together in the complex. For the highly disordered U4/U6.U5 tri-snRNP-specific proteins, high disorder content coupled with a high content of disorder with SS suggests a high potential for structure variability. We suggest that this potential is exercised upon the assembly and disassembly of the tri-snRNP. Among compositionally biased IDRs, only RS-like domains are commonly found in the late proteins. Between proteins of the U4/U6.U5 tri-snRNP, step 2 catalytic factors and the abundant B, B-act and C complex stage-specific proteins, we identified 12 RS-like IDRs, including a single RS-like IDR in the central part of the U4/U6 di-snRNP protein U4/U6-90K and the RS-like IDR on the N terminus of the U5 snRNP protein U5-100K [30]. The broad distribution of the RS-like IDRs leads us to propose that RS-like IDRs may be, in fact, a major driving force behind spliceosome dynamics in addition to fulfilling their role in the process of pre-mRNA recognition and intron/exon definition.

### Non-abundant proteins contain more compositionally biased disorder than core spliceosomal proteins

We repeated our IDR analysis for 122 additional proteins consistently found in the results of proteomics analyses of the major spliceosome (Table S1). The addition of these proteins increased the overall predicted disorder content of the major spliceosome proteome to 52.3%. Hence, the auxiliary spliceosomal proteins have their overall disorder content higher even than the core proteins.

**Figure 3:**
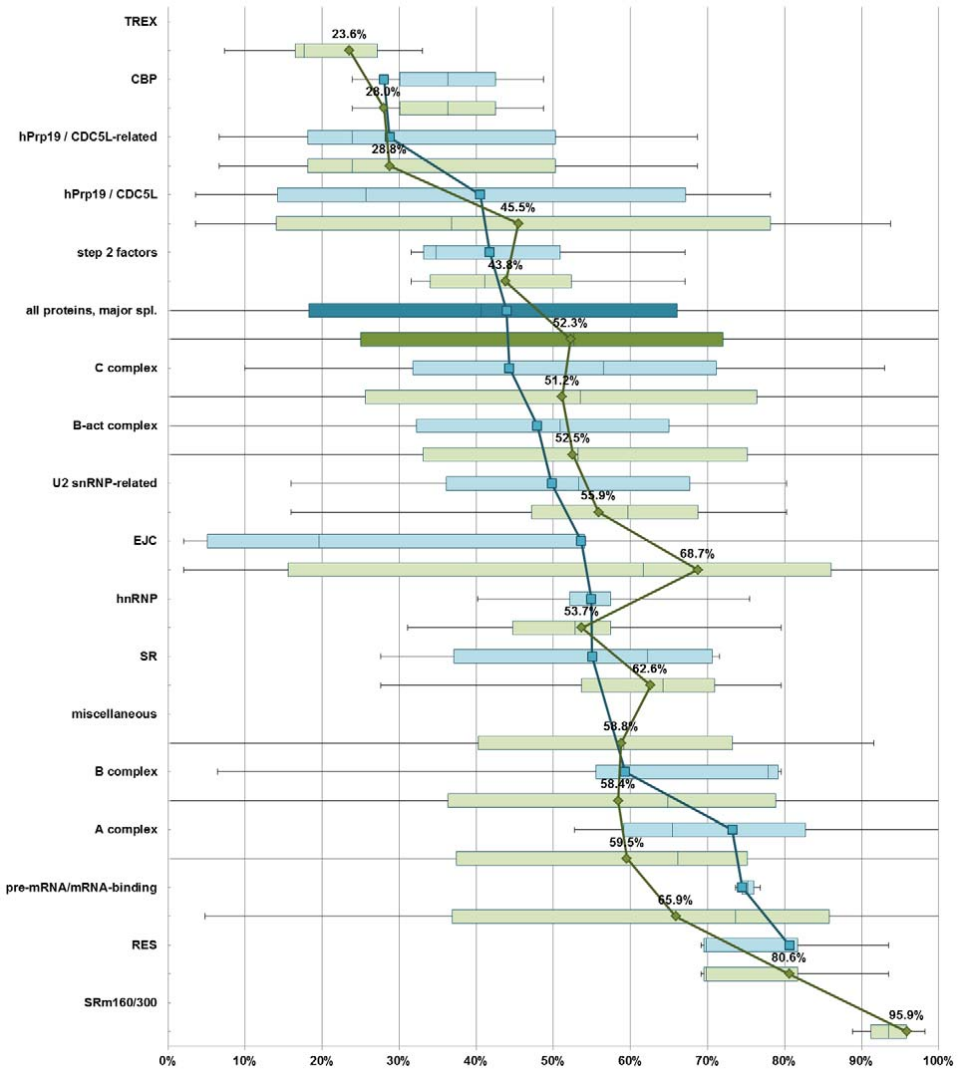
Disorder in core vs. non-abundant spliceosome proteins. Blue bars indicates values of intrinsic disorder content for core proteins, green bars for both core and additional spliceosome proteins. The blue and green lines indicate means for given protein groups, calculated per-residue. In deeper shade, values for all core (blue) and all (green) proteins associated with the major spliceosome.

For most protein groups, adding non-abundant proteins changed IDR content values by less than 10% of the respective lengths of proteins involved (Figure 3). In particular, non-abundant early (A-complex and B-complex-associated) proteins are, like abundant early proteins, estimated to be more disordered than B-act proteins and C-complex proteins (59.5% and 58.4% disorder content vs.52.5% and 51.2%). Compared to abundant proteins, non-abundant proteins are predicted to contain a larger amount of long regions of compositional disorder (Table S2). RS-like IDRs are again present in multiple proteins, including non-SR proteins. In the case of the EJC, three non-abundant proteins, acinus, pinin and RNPS1, supply the RS-like IDRs that are missing from the EJC as defined only by abundant proteins. We also found poly-P/Q regions, mainly in early (A-complex, U2 snRNP-related, pre-mRNA/mRNA-binding proteins and “miscellaneous” proteins) and hnRNP proteins. Short hnRNP-like G-rich regions are found predominantly in SR, A-complex, pre-mRNA/mRNA-binding proteins and “miscellaneous” proteins, as well as the EJC protein Aly/Ref. Most of the proteins that contain hnRNP-like G-rich IDRs have been confirmed to bind RNA. In short, the distribution of the non-hnRNP G-rich IDRs is similar to the distribution of other compositionally biased IDRs, and the distribution of compositionally biased IDRs in non-abundant proteins is similar to their distribution in abundant proteins.

Some auxiliary proteins, such as the two RS-like IDR-rich splicing coactivators SRm160/300, are both extremely long and extremely disordered (SRm300: 2752 residues, predicted 98.1% disorder content). In this particular case, the SRm160/300 proteins are thought to form a matrix promoting interactions between splicing factors [31].

### Compositionally biased disorder of spliceosome proteins (RS-like and glycine-rich) is associated with post-translational modifications (serine phosphorylation and arginine methylation)

We next considered the association of post-translational modifications (PTMs) of human spliceosomal proteins with intrinsic disorder. To do so, we compared our data on IDR distribution throughout the human spliceosomal proteome with PTM data from UniProt [32]. Four distinct PTMs are found in UniProt data in large enough numbers to warrant numerical analysis: phosphorylations (on various residues), lysine N-acetylations, other N-terminal acetylations and arginine methylations (various types). Of these, N-terminal acetylation is a ubiquitous cellular process not connected to splicing. 80–90% of human proteins are acetylated on the N terminus [33].

82.6% of all PTMs of spliceosomal proteins found in UniProt are phosphorylations (Table 1), of which phosphorylation on a serine is the most common (78.9% of all phosphorylations), followed by threonine (15.2%) and tyrosine (5.9%) phosphorylation. 32.2% of all phosphorylations are mapped to RS-like IDRs, even though such regions comprise only 7.1% of the combined length of the 252 spliceosome proteins. In the 122 core proteins of the major spliceosome, which include fewer SR proteins, RS-like IDRs comprise 3.2% of their combined length, but they encompass as many as 23.0% of all phosphorylation sites. This result suggests that the known cases of recorded functional importance of phosphorylation of RS-like IDRs in non-SR proteins may not be isolated, and that phosphorylation may be as important a control mechanism for the function of these sites as it is for the RS domains of SR proteins. 9.7% of PTMs are lysine N-acetylations, which map to ordered and disordered regions in proportions similar to the total amounts of order vs. disorder for both the core 122 and all 252 proteins (0.6:0.4 order vs. disorder),and therefore do not appear to be associated with either order or disorder. Finally, UniProt registers 74 cases of arginine methylations in the 252 spliceosome proteins (3.4% of all PTMs). Almost all sites of arginine methylation are located in hnRNP protein G-rich regions and shorter hnRNP-like G-rich regions in Sm proteins, SR proteins and A-complex, pre-mRNA-binding and miscellaneous RNA-binding proteins. Note that UniProt does not list any arginine methylations for some proteins, such as Sm-D3, that have been shown to contain methylated arginines [34] and where we found a G-rich region (Table S2). Hence, arginine methylations may be more widespread than indicated by database data. The consideration of arginine methylation has been so far overshadowed by the consideration of the far more widespread consideration of phosphorylation (see e.g. [8]). We suggest that the importance of arginine methylation for spliceosomal proteins should be considered in greater detail. In particular, the possibility exists that, if RS-like IDRs (of SR and other proteins) interact with the hnRNP-like G-rich regions (of hnRNP and other proteins), these interactions may be modulated by phosphorylation and by methylation. UniProt registers also six cases of lysine methylations at five unique residues, two of them in disordered regions and three in ordered regions. Five of the six cases occur in proteins with methylated arginines.

**Table 1:** Post-translational modifications in 252 spliceosome proteins.

Modification	Structural order	Disorder with SS	RS-like	Poly-P/Q	hnRNP-like G-rich	Noncharged	Charged	Other disorder	Total	Percent
Phosphorylation (*)	158	326	572	137	82	43	49	412	1779	82.6%
Lysine N-acetylation	127	30	12	4	6	0	3	27	209	9.7%
Other N-acetylation (**)	14	20	1	0	1	2	2	44	84	3.9%
Arginine methylations (***)	5	2	13	4	42	2	0	6	74	3.4%
Lysine methylations (****)	3	0	2	0	0	0	0	1	6	0.3%
Cysteine methyl ester	0	1	0	0	0	0	0	0	1	0.0%

(*) S,T and Y phosphorylation.

(**) N-terminal acetylation of MGASTV.

(***) Includes the keywords “dimethylarginine”, “asymmetric dimethylarginine”, “omega-N-methylarginine”.

(****) Includes the keywords “N6-methyllysine”, “N6, N6-dimethyllysine”, “N6, N6, N6-trimethyllysine”.

### ULMs are associated with early proteins, while other disordered recognition motifs are found throughout splicing complexes and candidate hub proteins are associated with later stages of splicing

To further analyze the possible roles of disorder that may acquire structure in the human spliceosome, we considered three sources of information: data from experimentally determined structures available in the Protein Data Bank (PDB) [35], predictions of disordered PFAM [36] domains and predictions of the most disordered proteins of the human spliceosome.

We browsed the experimentally determined structures of spliceosomal protein complexes to find out which regions predicted to be disordered in isolation were found to be ordered in a complex. Short disordered ligand peptides (<30 residues) that acquire structure upon binding larger partners are called Molecular Recognition Features (MoRFs) [37], while larger sequence features of this kind are called domain-length disordered recognition motifs [16]. In the structures of spliceosomal protein complexes, we found eight distinct regions that fit either definition (Table 2, Figure S3). Three of these regions were the previously defined ULMs (UHM Ligand Motifs), that is ligands for U2AF Homology Motif domains [38] (ELM database: LIG_ULM_U2AF65_1). Experimental structures containing ULMs represented U2 snRNP, U2 snRNP-related and A-complex proteins. Via a pattern recognition search, we found additional candidate regions for ULMs, mainly in low-abundance U2 snRNP-related proteins and A-complex proteins (Table S3). The majority of these tentative ULMs were predicted to be disordered. Although the presence of an individual ULM in a sequence may not be significant, we suggest that the concentration of sequences with ULM patterns at the early stage of the spliceosome action may be functionally relevant, and that the additional candidate ULMs may represent actual functional ULMs. If so, these additional ULMs could represent a non-essential extension of the essential UHM-ULM interactions, and UHM-ULM interactions may form an accessory network to the network created by compositionally biased IDRs (and their partners). Notably, a list of candidate UHM partners for ULMs also contains mainly early spliceosomal proteins [39].

**Table 2:** Regions predicted to be disordered, found to be ordered in experimentally solved complexes of spliceosomal proteins.

Region	Type	Protein	Region	Protein group	Partner (*)	Predicted ordered/disordered status in isolation	Structure	Reference
N-U1snRNP70_N	MoRF	U1-70K	8–22	U1 snRNP	U1-C (zf-U1)	disordered, next to ordered helix	3CW1	[29]
C-U1snRNP70_N	short, RNA-binding	U1-70K	63–89	U1 snRNP	U1 snRNA	disordered	3CW1	[29]
ULM (**)	MoRF	SF3b155	333–342	U2, SF3B	SPF45 (UHM)	disordered	2PEH	[87]
ULM	MoRF	U2AF65	90–112	U2 snRNP-related	U2AF35 (UHM)	disordered	1JMT	[38]
ULM	MoRF	SF1	13–25	A-complex (***)	U2AF65 (UHM)	disordered	1O0P	[88]
SF3b1	MoRF	SF3b155	377–415	U2, SF3B	SF3b14a/p14 (RRM)	partially ordered	2F9D	[89]
SF3a60_bindingd	Domain-length	SF3a60	71–106	U2, SF3A	SF3a120 (Surp)	partially ordered	2DT7	[90]
PRP4	Domain-length	U4/U6-60K	107–137	U4/U6 di-snRNP	U4/U6-20K	partially ordered	1MZW	[91]
PRP4 (****)	Domain-length	Prp18	77–115	step 2 factors		ordered	2DK4	
Btz	Domain-length	MLN51	169–196, 215–230	EJC	EIF4A3	disordered, next to ordered helix	2J0S	[92]

(*) Domain names in brackets.

(**) ULMs correspond to the ELM motif LIG_ULM_U2AF65_1, defined by the pattern [KR]{1,4}[KR]-x{0,1}-[KR]W-x{0,1}.

(***) Non-abundant A-complex protein.

(****) The PRP4 region of Prp18 is ordered and its structure in isolation was solved. It is included in the table since the PRP4 region of U4/U6-60K is predicted to be partially disordered.

Other recognition regions (U1snRNP70_N, SF3a60_bindingd, SF3b1, PRP4, Btz, all of which we labeled after PFAM regions) are found in complexes present at various stages of the splicing reaction. Notably, the U1snRNP70_N region encompasses two subregions, the C-terminal of which is the only predicted disordered region shown through an experimental structure to bind RNA. Via a profile search, we found two additional candidate regions for the Btz motif and one additional candidate PRP4 region. The candidate Btz regions are found in TRAP150, an abundant A-complex protein, and its paralog BCLAF1, a low-abundance pre-mRNA/mRNA-binding protein that has been implicated in a wide range of processes [40]. The candidate PRP4 region is found in the U2 snRNP SF3A protein SF3a66. Unlike the ULMs, which appear to be widespread and function in multiple contexts at the early stage of splicing, non-ULM motifs appear to have specific functions and bind specific partners.

To find other potential domain-length recognition motifs in spliceosomal proteins, we considered the PFAM domains that mapped to predicted IDRs. We found 51 such PFAM domains (Table S4), which included both conserved disordered regions in otherwise ordered proteins and the only conserved regions of almost completely disordered proteins. We propose these domains as targets for experimental structural analyses.

Notably, when we compared the list of disordered PFAM domains with the list of the most disordered proteins in the spliceosomal proteome, we found that this group includes two out of three U4/U6.U5 tri-snRNP-specific proteins (U4/U6.U5-27K and 110K), as well as several conserved proteins associated with the B, B-act and C complex (e.g. MFAP1, RED, GCIP p29) that are also abundant in the human spliceosomal proteome [4] (Table 3; Figure S4). We suggest that the presence of conserved motifs comprising disordered PFAM domains in these abundant conserved highly disordered proteins may allow them to act as “hub” proteins. If so, these proteins may be crucial to spliceosome dynamics. Targeted deletions of the conserved motifs within these proteins may help elucidate their role.

**Table 3:** “Most highly disordered” proteins in the spliceosomal proteome.

Abundance	Protein	Disorder fraction	PFAM domains	Group
Abundant	SPF30	80.3%	SMN	U2 snRNP-related
	U4/U6.U5-110K	87.9%	SART-1	U4/U6.U5 trisnRNP
	U4/U6.U5-27K	76.8%	DUF1777	U4/U6.U5 trisnRNP
	CCAP2	78.2%	Cwf_Cwc_15	hPrp19/CDC5L
	TRAP150	100.0%		A-complex
	MFAP1	79.3%	MFAP1_C	B-complex
	RED	79.5%	RED_N, RED_C	B-complex
	MGC23918	100.0%	cwf18	B-act complex
	HSPC220	84.8%	Hep_59	C-complex
	GCIP p29	93.0%	SYF2	C-complex
Non-abundant	U11/U12-59K	91.1%		U11/U12
	Npw38BP	93.8%	Wbp11	hPrp19/CDC5L
	MLN51	100.0%	Btz	EJC
	pinin	92.3%	Pinin_SDK_N, Pinin_SDK_memA	EJC
	MGC13125	93.5%	Bud13	RES
	C19orf43	88.6%		A-complex
	FLJ10154	100.0%		A-complex
	CCDC55	100.0%	DUF2040	B-complex
	CCDC49	100.0%	CWC25	B-complex
	PRCC	100.0%	PRCC_Cterm	B-act complex
	DGCR14	86.1%	Es2	C-complex
	DKFZP586O0120	100.0%	DUF1754	C-complex
	FLJ22626	100.0%	SynMuv_product	C-complex
	LENG1	100.0%	Cir_N	C-complex
	BCLAF1	100.0%		pre-mRNA/mRNA-binding

Entries in this table fulfill simultaneously two conditions: they have a predicted disorder content >75%, and do not contain any PFAM domains that correspond to ordered structural domains.

### Conserved disordered regions in spliceosomal proteins are less widespread and evolutionarily younger than essential ordered domains in the core of the spliceosome

As spliceosomal proteins found in human are typically conserved throughout eukaryotes [41], we used the set of proteins found in the human spliceosomal proteome to determine the evolutionary path for the accumulation of order and disorder in the spliceosomal proteome. We investigated whether conserved ordered and disordered PFAM domains present in human spliceosomal proteins were present in the last eukaryotic common ancestor species (LECA), according to [42], and whether they are currently ubiquitous outside of eukaryotes.

The majority of both ordered and disordered PFAM domains were present in LECA (Table 4). However, while almost none of the disordered domains are currently widespread in prokaryotes, at least one-third of the ordered domains are. This suggests that, unlike disordered domains, these ordered domains may have been transferred to eukaryotes from prokaryotes, and may be, in fact, older than LECA. Notably, the contribution of these evolutionarily old domains is much higher in the ordered regions of the snRNP proteins than in the general group of abundant proteins. As many as 19 out of 29 (distinct) domains of the U4/U6.U5 tri-snRNP are “old” domains. Furthermore, the majority of the proteins of the U4/U6.U5 tri-snRNP, including the Sm/Lsm proteins but not the U4/U6.U5 tri-snRNP-specific proteins, either possess homologs among bacterial and non-splicing-related eukaryotic proteins or are composed of ubiquitous domains [1], [43] (Table S5). The U5 snRNP contains ordered domains similar to those present in maturase proteins of modern bacterial group II introns [44], from which the spliceosome snRNAs and introns are predicted to have evolved [45]. In consequence, this group of proteins/domains as has a strong potential to evolutionarily predate the eukaryotes. Likewise, the C-terminal region of the splicing helicases hPrp2/22/16/43 is also found in some bacterial helicases such as the *Escherichia coli* HrpA and therefore is likely to be ancient [46]. We suggest that the spliceosome likely accrued piecewise, and that these evolutionarily old regions, which are also the most ordered regions of the spliceosome, were recruited into the system first and formed the structural and functional core of the spliceosome. Disordered regions, as well as ordered domains only found in eukaryotes, would in this scenario appear in the spliceosome later.

**Table 4:** Statistics of conserved ordered and disordered PFAM domains.

	ordered domains			disordered domains		
	all proteins	abundant proteins	U4/U6.U5 tri-snRNP (*)	all proteins	abundant proteins	U4/U6.U5 tri-snRNP
all domains	124	86	29	46	24	5
domains found in LECA	121	86	29	36	22	5
domains found in prokaryotes (**)	47 (37.9%)	34 (39.5%)	19 (65.5%)	1 (0.0%)	0 (0.0%)	0 (0.0%)

(*) Including the LSM domain present in Sm and Lsm proteins.

(**) In >100 copies.

### The spliceosomal and the ribosomal proteomes have a similar fraction of disordered residues, but different types of intrinsic disorder

As the final step of our analysis, we compared the fractions and distributions of intrinsic disorder in the proteomes of the subunits of the human major spliceosome and the human and the *Escherichia coli* ribosomes. The bacterial ribosome was chosen to supplement structural information on disorder-to-order transition, as no crystal structure of the human ribosome is presently available.

Our comparison revealed a number of similarities and differences between the proteins of the human snRNP subunits and both ribosomes (Table 5).The percentage fraction of residues predicted to be disordered is slightly higher in the ribosomal proteins compared to proteins of the spliceosomal snRNP subunits. The human ribosome contains more intrinsic disorder than the *E. coli* one, in keeping with the overall higher disorder content in eukaryotic proteins [47]. However, the types of the predicted disorder in the ribosomes and in the spliceosome are different. IDRs in ribosomal proteins are much shorter. While the number of proteins with at least one IDR≥30 residues are similar between the human ribosome and the human spliceosome, the spliceosome subunits contain twice as many proteins with at least one IDR≥70 residues as the human ribosome (Figure S5). Furthermore, the majority of intrinsic disorder in ribosomal proteins is predicted to contain SS elements, while the majority of intrinsic disorder in spliceosomal snRNP proteins is predicted not to contain secondary structure. There are 15 distinct non-SS IDRs≥70 residues in the subunits of the human spliceosome, but only three such regions in the human ribosome and none in the bacterial ribosome. Disordered regions ≥70 residues without secondary structure comprise 8.3% of the total mass of the snRNP subunits of the major human spliceosome, but only 0.4% in the human ribosome (Figure S6). Hence, intrinsic disorder in the ribosomes is considerably more “structured” than the disorder in the spliceosome. Both in the *E. coli* and in the human ribosomes, the large subunit is predicted to contain higher percentage of disorder than the small subunit. However, the differences in the fraction and type of disorder are less pronounced between the ribosomal subunits than between the various subunits of the spliceosome. The ribosome is therefore more homogeneous with respect to the distribution of the intrinsic disorder of its proteins than the spliceosome.

**Table 5:** Features of intrinsic disorder in *E. coli* and human ribosomes and human major spliceosome snRNP subunits.

Feature	Ribosome, *E. coli*	Ribosome, human	Major spliceosome, snRNP subunits, human
Number of proteins	54	80	45
Maximum protein length (aa)	557 (S1)	427 (L4)	2335 (U5-220K/hPrp8)
Mean protein length (aa)	132	170	453
Fraction of predicted disorder (% of the combined lengths of proteins)	37.7%	47.0%	34.1%
Number of proteins with at least one IDR ≥30 residues	28	61	28
Number of proteins with at least one IDR ≥70 residues	1	19	23
Mean IDR length (aa)	28	39	93
Fraction of predicted disordered residues with secondary structure (% predicted disorder)	66.6%	64.0%	41.9%
Number of non-PSE IDRs ≥70 residues	0	3	15
Fraction of predicted disordered residues found in the crystal structure of the complex (% of predicted disorder)	98.9%	—	<10% (U1 snRNP)
Minimal and maximal fractions of predicted disordered residues for individual subunits	34.8% (small subunit) - 40.0% (large subunit)	39.1% (small subunit) - 52.2% (large subunit)	20.1% (U5 snRNP) - 65.5% (U1 snRNP)
Maximum RNA length (nt)	2904 (23S)	5070 (28S)	188 (U2 snRNA)(*)
RNA fraction of total weight (% total weight)	65.2%	60.3%	8.2%

(*) *Saccharomyces cerevisiae* U1 snRNA is 570 nts long, while the U2 snRNA is 1172 nts long. Such exceptional lengths are restricted to the genus *Saccharomyces*.

The inspection of crystal structures confirms the predicted differences. 98.9% of predicted disordered residues of 51 *E. coli* ribosomal proteins are found ordered in one or more crystal structures of this ribosome. Only three proteins, L10, L7/L12 and S1, are missing from all crystal structures of ribosomes deposited in the PDB. Of these proteins, only L7/L12 contains an interdomain linker that is confirmed not to acquire structure in a complex [48], while only S1 contains a C-terminal disordered extension whose fate in a ribosome-bound form is unknown. This contrasts with the experimentally determined structure of the U1 snRNP, which reveals order for less than 10% of residues predicted to be disordered in isolated U1 proteins.

As described in the Introduction, the main function fulfilled by IDRs in the ribosome is to be the “mortar” that fills in the gaps in the rRNAs, while the RNA forms the bulk of the macromolecular structure of the ribosome and defines its shape and catalytic center [23], [49]. Only in few cases is a different function realized. For instance, the flexible interdomain linker of protein L7/L12 interfaces the ribosome with ribosome-acting GTPases [48]. We suggest that the prominence of the “mortar” function is the reason both for the greater homogeneity of disorder types and their spatial distribution in the ribosomes, and the prevalence of disorder with SS in the ribosomes.

Although, in percentages, both the ribosomes and the spliceosome contain a similar amount of SS disorder, so far, there is very little structural evidence for the “mortar” function of the proteins of the spliceosome. We found only one predicted disordered region confirmed to bind RNA in all experimental structures of the spliceosome (C-terminal part of the U1snRNP70_N region, Table 2). Most experimental structures of splicing-related complexes feature ordered domains on the protein side. It is possible that novel structures will reveal binding interfaces wherein protein disorder supports the RNA in a “mortar”-like manner. However, the “mortar” role of intrinsic disorder may be simply less important in the spliceosome. The ribosomal RNA is longer in residues than any given ribosomal protein, occupies more space and has a higher molecular mass than all ribosomal proteins combined (Figure S6). In comparison, the snRNAs are much shorter than the rRNAs. Being shorter, they may be more likely to form a catalytically active form unaided by proteins and thus be in less need of “mortar”.

### Summary and conclusions

The spliceosome has been called a “molecular machine” [11]. While useful, this metaphor may also be misleading, as it brings to mind the image of a precise, assiduously controlled and operated mechanism proceeding to perform the splicing reaction according to discrete and precise steps. This mechanistic point of view of the spliceosome action leaves very little space to uncertainty, randomness, and fuzziness.

In this work, we made multiple predictions regarding individual regions of human spliceosomal proteins as well as systematically analyzed the fraction, distribution and types of disorder across the various spliceosomal components. Summarizing, we found that the spliceosome, far from being a uniformly ordered machine, can be divided into three layers:

- An inner layer, which best fits the definition of a “machine”. It includes the ordered cores of U2 snRNP SF3B, U4/U6 di-snRNP and U5 snRNP, as well as the Sm proteins of U1 snRNP and ordered C termini of the catalytic helicases. This layer also includes snRNAs. Proteins from this layer mainly assist the catalysis of the splicing reaction, and publications regarding this layer stress relatively precise mechanisms, such as kinetic proofreading [50]. Sm proteins, ordered proteins of the U4/U6 di-snRNP and U5 snRNP, as well as the C termini of catalytic helicases, are most likely the evolutionarily oldest peptide elements of the spliceosome.
- A middle layer, which is associated mostly with “structured” disorder (disorder with SS). It contains an abundance of domain-length disordered recognition motifs, disorder with predicted secondary structure that can act as, e.g., preformed structural elements and/or dual personality disorder, and long, highly disordered proteins with conserved disordered regions. Spatiotemporally, this layer is associated with U4/U6.U5 tri-snRNP-specific proteins, and B, B-act and C-complex non-snRNP proteins. Functionally, this layer is associated with spliceosome assembly, catalytic activation and dynamics. Many of these regions are phosphorylated. In addition to disorder with SS, this layer is also associated with some RS-like IDRs that function in splicing dynamics, such as [30]. This layer is also associated with ubiquitin-dependent systems. Ubiquitin has been shown to control the dynamics of the spliceosome in several cases [51]. Proteins of the spliceosome contain many ubiquitin-related domains, and the majority of these domains are found in the proteins associated with the later stages of splicing [52].
- An outer layer, which is associated with mostly “unstructured” disorder. It is enriched in regions of long, compositionally biased disorder that may function as sensors that the spliceosome extends to the surrounding environment. These regions contain interaction sites such as RS-like IDRs, hnRNP-like G-rich regions, polyproline regions and ULMs. They may interact with each other, or with small ordered structural domains such as the Tudor domain (bound by hnRNP-like G-rich regions) and GYF domain (bound by polyproline regions). On the other hand, small RNA-binding domains present in this layer, such as RRM (RNA Recognition Motif) and PWI, may aid in the binding of the substrate pre-mRNA. The function of this layer is regulated by phosphorylation (e.g. in RS-like IDRs) and methylation (e.g. in hnRNP-like G-rich regions). Spatiotemporally, this layer is associated with early (A-complex, U1, U2 SF3A, U11/U12, U2-related) proteins, with SR, hnRNP proteins, and SRm160/300 proteins, and with RES complex proteins. Functionally, this layer is associated with early recognition, intron/exon definition, and alternative splicing regulation processes.

Full understanding of spliceosome activity requires information about each of its elements, at different functional stages [11]. Our predictions provide a number of testable functional hypotheses:

- We provide the proteins and positions of all types of compositionally biased disordered regions in spliceosomal proteins. Based on the colocation of two types of disordered regions (RS-like and G-rich), we suggest that these regions may interact with each other. As these two types of disordered regions are found in multiple proteins throughout the human spliceosomal proteome, we also suggest the possibility that many more human spliceosomal proteins interact nonspecifically with each other and the RNAs than previously suggested. Large-scale deletions of compositionally biased regions may suggest essential subsystems of this interaction network;
- We found that arginine methylation in spliceosomal proteins is associated with intrinsically disordered regions. We also suggest that arginine methylation and serine phosphorylation act in step to regulate the interaction network based on compositionally biased disordered regions. The elucidation of the effect of post-translational modifications, such as conformational transitions and molecular interactions that depend on the introduction or removal of particular modifications, can also lead to an improved understanding of regulatory mechanisms;
- We provide candidate ULM sequences that can bind known and predicted UHM domains throughout the early stages of splicing. These sequences may participate in the regulation of particular instances of splicing;
- We suggest several abundant conserved proteins found in the later stages of splicing that may function as “hub” proteins (e.g. MFAP1, GCIP p29, U4/U6.U5 tri-snRNP proteins). Targeted deletions of ordered motifs within these proteins may reveal regions responsible for the formation of particular spliceosomal complexes, their rearrangements, and interactions with regulatory factors.

Our prediction that more than one-third of the residues of the snRNPs are disordered has significant implications for the structural studies of the spliceosome. While much progress has been achieved in the determination of global shapes of various spliceosomal assemblies by cryoEM [53], experimental structural information is missing for many regions of spliceosomal proteins. Intrinsic disorder in the spliceosome explains why: the functional importance of disordered regions notwithstanding, their physico-chemical properties make them notorious spoilers of crystallization experiments [54]. Our predictions of disorder may guide the preparation of protein variants for crystallization that should be limited to regions that are intrinsically ordered or at least predicted to become ordered upon complex formation. For long disordered regions without secondary structure, stable conformations may not be obtained even in complexes. However, the structural characterization of intrinsically disordered elements of the spliceosome may require the application of completely different methods, such as small angle X-ray or neutron scattering (SAXS or SANS) experiments (review: [55]) and modeling with computational tools such as the Ensemble Optimization Method [56]. The results of our analyses will hopefully aid these efforts.

## Methods

### Data

Spliceosome proteins with GI identifiers supplied in Table S1 were downloaded from the NCBI Protein database. Protein names and identifiers were acquired from [4], [6], [7], [57]–[61]. Division into abundant and non-abundant proteins was based on [4]. Assignment into protein groups was based mainly on [4], aided by information from: [6], [58]–[60]. “Miscellaneous” proteins were classified in primary sources, variably, as “miscellaneous proteins”, “miscellaneous splicing factors”, “additional proteins”, “proteins not reproducibly detected”, “proteins not previously detected”.

### Prediction of intrinsic disorder and binding disorder

Initial predictions of intrinsic disorder were carried out using the GeneSilico MetaDisorder server (http://iimcb.genesilico.pl/metadisorder/; [24]). Subsequently, disorder boundaries yielded by MetaDisorder were corrected manually based on predictions of secondary structure and solvent accessibility yielded by the GeneSilico MetaServer gateway (https://genesilico.pl/meta2/; [25]). In particular, sequence regions predicted to exhibit stable secondary structure and high fraction of solvent inaccessible residues, and confidently aligned to experimentally determined globular protein structures, were considered ordered regardless of the primary disorder prediction. Prediction of binding disorder was carried out using the ANCHOR server [62].

### Assignment of disorder with predicted secondary structure

In disorder with SS, the disordered region is predicted to contain one or both types of canonical α and β SS elements. The predicted secondary structure may be either pre-formed in the disordered state or appear only upon the formation of a stable structure, e.g. upon binding to another molecule. This type of disorder also at times contains short ordered regions (Table 6, Figure S7).

**Table 6:** Features of different IDR classes in the 130 spliceosomal proteins.

IDR class	Description	Number of regions	Mean length	Compositional bias
disorder with SS	contains secondary structure	95 (predicted to contain coiled coils), 115 (other types)	64 aa (predicted to contain coiled coils), 55 aa (other types)	RKDE with additional MQW (predicted to contain coiled coils), no rule (other types)
compositionally biased, RS-like	biased towards arginine and serine residues	35	65 aa	RS
compositionally biased, polyP/Q	noncharged with poly P/Q (P/Q(n), n≥3)) repeats	17	138 aa	PQMGVWA
compositionally biased, hnRNP G-rich	contains RGG and related repeats ([RSY]GG, R[AGT][AGTFIVR]) (*)	4 (hnRNP proteins), 10 (other proteins)	145 aa (hnRNP proteins), 56 aa (other proteins)	GRY
compositionally biased, noncharged	biased towards noncharged residues	16	45 aa	PQMGVWA
compositionally biased, charged	biased towards charged residues	9	57 aa	RKDE

(*) [72]: XGG, where X aromatic or long aliphatic; arginine methylation data: R[AGT][AGTFIVR].

We defined regions of disorder with SS (predicted intrinsic disorder with predicted secondary structure elements) as regions for which simultaneously the majority of intrinsic disorder prediction methods on the MetaServer gateway yielded predictions of disorder and the majority of secondary structure prediction methods yielded predictions of secondary structure elements. Multiple closely spaced secondary structure elements (connected by loops <20 residues) in a predicted disordered region were treated as elements of a single IDR with SS. If an IDR was predicted to contain α-helical elements and coiled-coil prediction methods aggregated on the MetaServer also yielded a prediction, the IDR was classified into the special class of disorder with coiled coils.

### Assignment of disorder with compositional bias

In compositionally biased disorder, the amino acid composition of the region deviates highly from the usual. We estimated compositional bias based on the absolute frequencies of occurrence of residues, compared to their usual frequency in vertebrates, as reported on the website http://www.tiem.utk.edu/~gross/bioed/webmodules/aminoacid.htm (information from [63], [64]). A residue was considered overrepresented if (a) the region under consideration displayed considerable compositional bias (at least one kind of residue occurred with a frequency >20% or five times higher than its usual frequency of occurrence in vertebrates) and (b) this particular residue occurred in the region with a frequency >20% or three times higher than the usual frequency of occurrence in vertebrates.

For several types of compositionally biased IDRs with a previous description in literature, we sought to define relevant standard IDR subclasses within our classification (Table 6):

- RS-like: IDRs that are rich in arginine and serine residues. These regions were shown to be intrinsically disordered [65]. They are predicted to have high solvent accessibility (Figure S7). They may be phosphorylated on the serines [66]. RS-like regions were found in splicing factors from the SR family (“RS domains”) and in other spliceosomal proteins [67]. RS domains of SR proteins bind other RS-like IDRs as well as (pre-m)RNA and are crucial for the establishment of a network of weak contacts at the initial stages of splicing and intron/exon definition [66]. Phosphorylation of some RS domains enhances their binding [68], [69]. Phosphorylation of the RS-like IDR of the U5 snRNP protein DDX23 is also required for its stable association (with the U4/U6.U5 tri-snRNP) [30].
- polyP/Q: IDRs that contain repeats of proline or glutamine residues. polyP/Q regions are capable of generating type II poly-P or poly-Q helices [70] and may contain short linear motifs involved in nonspecific binding of GYF and WW-type domains [41]. They are predicted to have high solvent accessibility (Figure S7). Several spliceosomal proteins, such as the Sm protein SmB/B', were shown to contain polyP/Q regions that interact with GYF and WW-type domains. Collectively, these regions are necessary for the formation of complex A [71].
- hnRNP-like G-rich: IDRs that contain RGG and related repeats ([RSY]GG, R[AGT][AGTFIVR]) that can be classified as short (≤100 residues) and long ones. These regions are predicted to have low solvent accessibility (Figure S7), but do not contain canonical higher order structures [72]. Repeats that contain arginines may be methylated on these residues [73]. Long G-rich IDRs were found in hnRNP proteins [74], while shorter G-rich IDRs are found in other splicing proteins, such as SmB/B', SF2/ASF and U1-70K ([73], [75], [76]). The G-rich region of hnRNP A1 has been shown to bind *in vitro* itself and other hnRNP proteins [77], to be necessary for the binding of hnRNP A1 to the U2 and U4 snRNPs [78], and to silence splicing [79]. Arginine-methylated G-rich regions may interact with the Tudor domain of the SMN protein [80], [81]. Arginine methylation of yeast U1-70K homolog decreases binding of this protein by protein Npl3 [76].

We also developed two additional subclasses of compositionally biased IDRs to complement these classes of compositionally disordered IDRs:

- “noncharged” disorder, which is rich in noncharged residues (PQMGVWA);
- “charged” disorder, which is rich in charged residues (RKDE). The “charged” compositionally biased disorder is similar to a type of disorder with SS that has predictions for coiled-coil secondary structure.

### PTM data

Site identifiers of 2153 known or possible post-translational modifications, including 720 modifications of the 122 core proteins, were downloaded from UniProt [32]. The following post-translational modifications were included: serine-, threonine- and tyrosine phosphorylations, lysine N-acetylations, N-alpha-terminal N-acetylations of non-lysine residues (MGASTV), various arginine methylations and various lysine methylations. All site identifiers available were used in the analysis (i.e. including sites with a status note “By similarity” and sites identified as “Potential” or “Probable”). 132 modification sites had a status note “Status = By similarity” and 8 had a status note “Status = Potential” or “Status = Probable”. Removing sites identified “By similarity” and sites identified as “Potential” or “Probable” did not impact overall statistics. In the listing, different modifications at same residues are considered separately (e.g. different possible arginine methylations), and the paper follows this model.

### Pattern recognition and motif search

Assignment of boundaries for hnRNP-like G-rich regions and for positions of candidate ULMs was based on pattern analysis. For hnRNP-like G-rich regions, the following patterns were used: [RSY]GG-x{1,50}-[RSY]GG-x{1,50}-[RSY]GG; R[AGT][AGTFIVR]-x{1,25}-RGG-x{1,25}-R[AGT][AGTFIVR]. For ULMs, the following pattern was used: [RK]{1,}-[RK]-x{0,1}-[RK]{1,}-x{0,1}-W-x{0,2}-[DE]{1,}. The ULM consensus pattern was based on the sequences of known ULMs found in experimentally determined structures of ULM complexes. This stringent pattern does not retrieve all of the *bona fide* ULMs in protein SF3b155 that display a weaker binding affinity to the U2AF65 partner than the ULM found in the experimentally determined structure [82]. We decided to use a stringent pattern in order to reduce the number of possible false positives compared to the more lenient pattern described in literature [39]. Search for domain-length disordered recognition motifs was carried out with HHSEARCH [83].

### Assignment of PFAM domains in disordered regions and LECA presence for disordered PFAM domains

PFAM IDs were assigned on the PFAM website [36]. The list of disordered domains present in LECA was established based on a list of predicted LECA domains kindly provided by Prof. Adam Godzik and Dr. Christian M. Zmasek [42].

### Analysis of disorder and disorder-to-order transition in *E. coli* and human ribosome

*E. coli* and human ribosomal proteins were extracted from the Ribosomal Protein Gene database (RPG) [84]. The following crystal structures of *E. coli* ribosomes and ribosomal proteins were used to determine disorder-to-order transitions: majority of proteins: PDB ID: 2QAM (subunit 50S, resolution 3.21 Å) and 2QAN (subunit 30S, resolution 3.21 Å); protein L31: ribosomal structure 2AW4; protein L1: ribosomal structure 3FIK. For protein L7/L12, a dimer structure was used (PDB ID: 1RQU), while for protein S1 only the one available structure of a single domain was used (PDB ID: 2KHI).

Although a crystal structure of a eukaryotic ribosome has been recently determined, many amino acid residues within this structure are unassigned [85]. Hence, this structure is unsuitable for the examination of sequences that alter their state between order and disorder.

### Visualization

Disorder and binding disorder plots were generated using the ANCHOR server (http://anchor.enzim.hu) [62]. Molecular structure graphics were produced with UCSF Chimera [86].

## Supporting Information

Figure S1 **The hierarchy of classification of intrinsic disorder in the spliceosomal proteome.** “Compositionally biased disorder” includes only disorder predicted not to contain any secondary structure elements.
(TIF)

Figure S2 **Types of disorder in core spliceosomal proteins.** This figure shows the fractions of all types of disorder with SS (left) and compositionally biased disorder (right) in various groups of core spliceosomal proteins. Values are given as fractions of total disorder. In this figure, disorder with SS is divided based on the presence or absence of coiled coils and types of secondary structure.
(TIF)

Figure S3 **MoRFs in the structures of spliceosome proteins.** A: N-U1snRNP70_N (in yellow) and C-U1snRNP70_N (in red) (protein U1-70K in the structure of U1 snRNP with removed Sm proteins, PDB ID: 3CW1). B: ULM (protein SF3b155 in complex with SPF45, PDB ID: 2PEH). C: ULM (protein U2AF65 in complex with U2AF35, PDB ID: 1JMT). D: SF3b1 (protein SF3b155 in complex with SF3b14a/p14, PDB ID: 2F9D). E: SF3a60_bindingd (protein SF3a60 in complex with SF3a120, PDB ID: 2DT7). F: Btz (protein MLN51 in the structure of the exon-junction complex, PDB ID: 2J0S).
(TIF)

Figure S4 **Disorder plots for highly disordered spliceosome proteins.** Example disorder plots created by the ANCHOR server, http://anchor.enzim.hu. Red line: disorder probability; blue line: probability of binding another molecule at the residue; blue line at the bottom: another representation of the binding probability (the darker the blue, the higher the probability). A. MLN51 (EJC protein). The region corresponding to the Btz MoRF lies between residues 169–230. B. U4/U6.U5-110K. C. U4/U6.U5-27K.
(TIF)

Figure S5 **IDR lengths in** ***E. coli*** **and human ribosome and human major spliceosome snRNP subunits.** This graph shows the fraction of proteins in the proteomes of the *E. coli* (orange) and human ribosome (green) and the snRNP subunits of the major spliceosome (blue) that contain at least one IDR of a given length.
(TIF)

Figure S6 **Structural regions in** ***E. coli*** **and human ribosome and human major spliceosome snRNP subunits.** This graphs shows the fractions of the total weight of the three complexes taken up by different types of structural regions. The Sm proteins were calculated four times each towards the weight of the spliceosome.
(TIF)

Figure S7 **Disorder plots for various types of IDRs found in spliceosome proteins.** Example disorder plots created by the ANCHOR server, http://anchor.enzim.hu. Red line: disorder probability; blue line: probability of binding another molecule at the residue; blue line at the bottom: another representation of the binding probability (the darker the blue, the higher the probability). A. IDR with SS: SF3b145, residues 738–818; B. RS-like IDR: protein 9G8, residues 121–215; C. polyP/Q IDR: SF3a66, residues 216–307; D. hnRNP G-rich IDR: hnRNPA1, residues 200–285. Interpretation of the plots: A is predicted to contain short regions of order in regions of disorder, B and C are predicted to be almost completely unfolded in isolation and D is largely insoluble. A, B and C contain regions predicted to be binding. In the case of the RS region, this encompassed almost its entire length.
(TIF)

Table S1 **Proteins of the human spliceosomes divided into groups.**
(XLSX)

Table S2 **Compositionally biased regions of spliceosome proteins.**
(XLSX)

Table S3 **Candidate ULMs, Btz and PRP4 regions in spliceosomal proteins.**
(XLSX)

Table S4 **PFAM domains that map to disordered regions in human spliceosomal proteins.**
(XLSX)

Table S5 **Conserved ordered regions in the core of the human spliceosome.**
(XLSX)
