# Supplementary figures and images for: Intrinsic Disorder in the Human Spliceosomal Proteome
Source: PLoS Comput Biol. 2012 Aug 9;8(8):e1002641. doi: 10.1371/journal.pcbi.1002641 (PMC3415423; doi:10.1371/journal.pcbi.1002641)

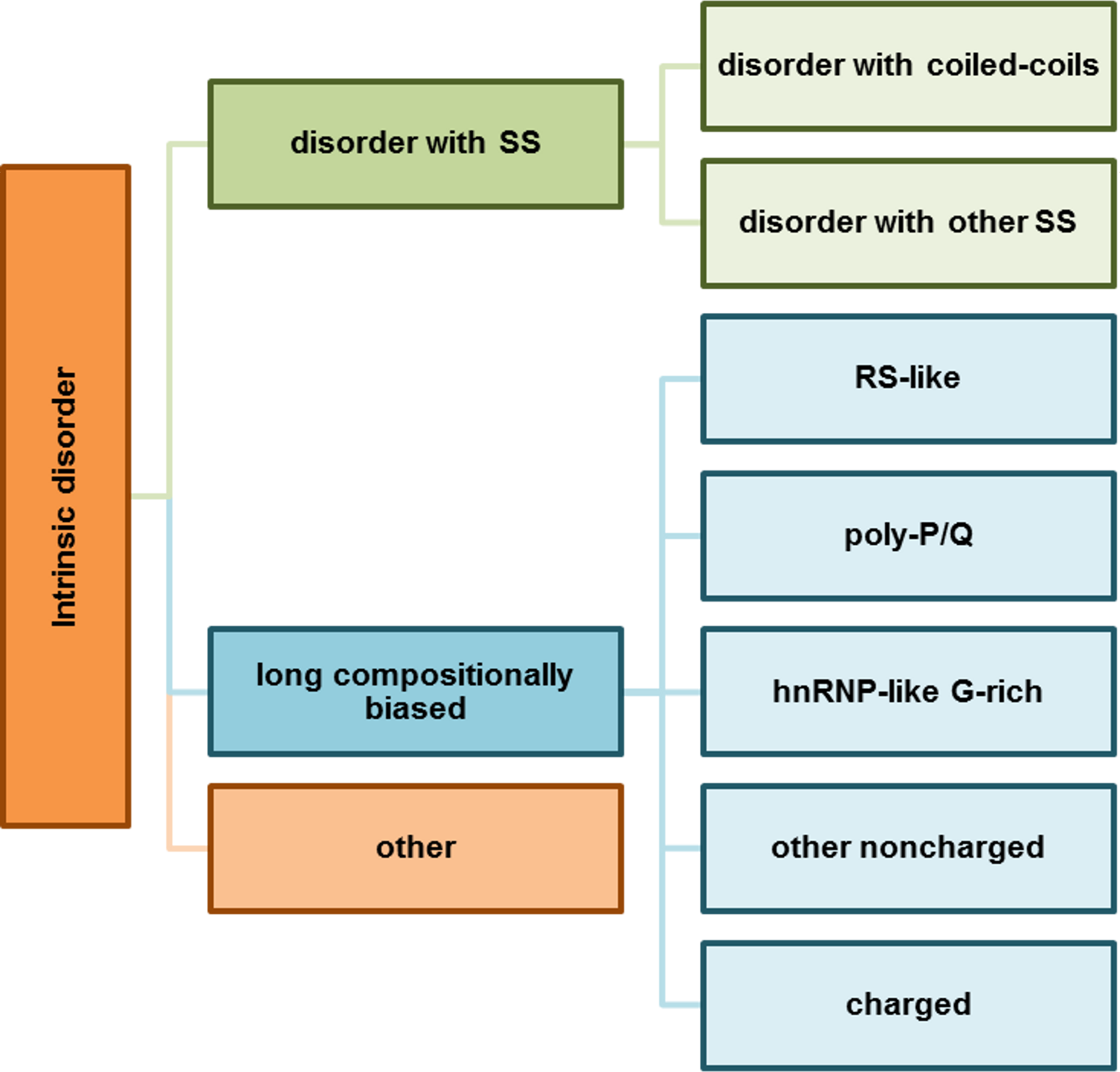

Supplement: Figure S1 — The hierarchy of classification of intrinsic disorder in the spliceosomal proteome. “Compositionally biased disorder” includes only disorder predicted not to contain any secondary structure elements. (TIF) [file pcbi.1002641.s001.tif]

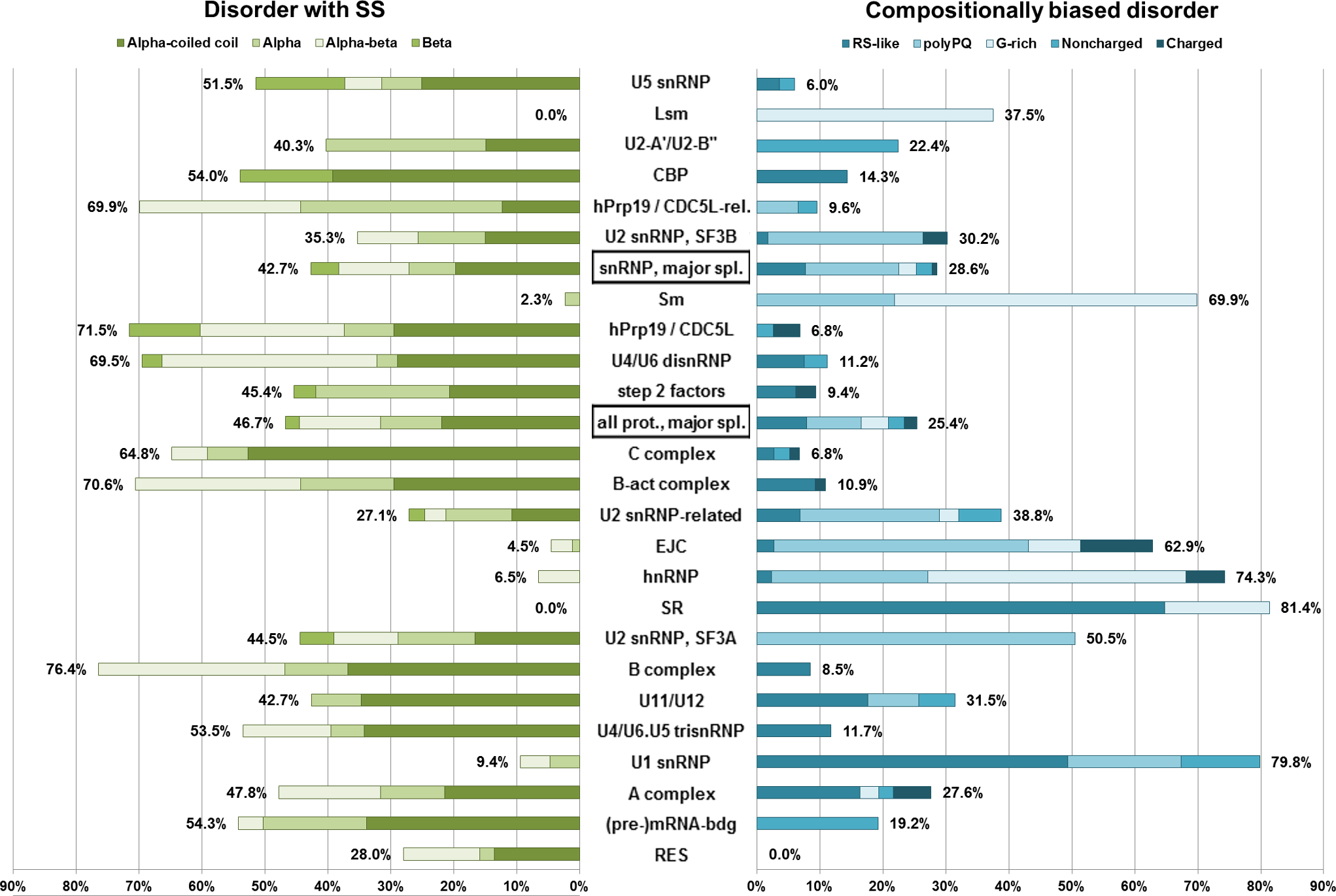

Supplement: Figure S2 — Types of disorder in core spliceosomal proteins. This figure shows the fractions of all types of disorder with SS (left) and compositionally biased disorder (right) in various groups of core spliceosomal proteins. Values are given as fractions of total disorder. In this figure, disorder with SS is divided based on the presence or absence of coiled coils and types of secondary structure. (TIF) [file pcbi.1002641.s002.tif]

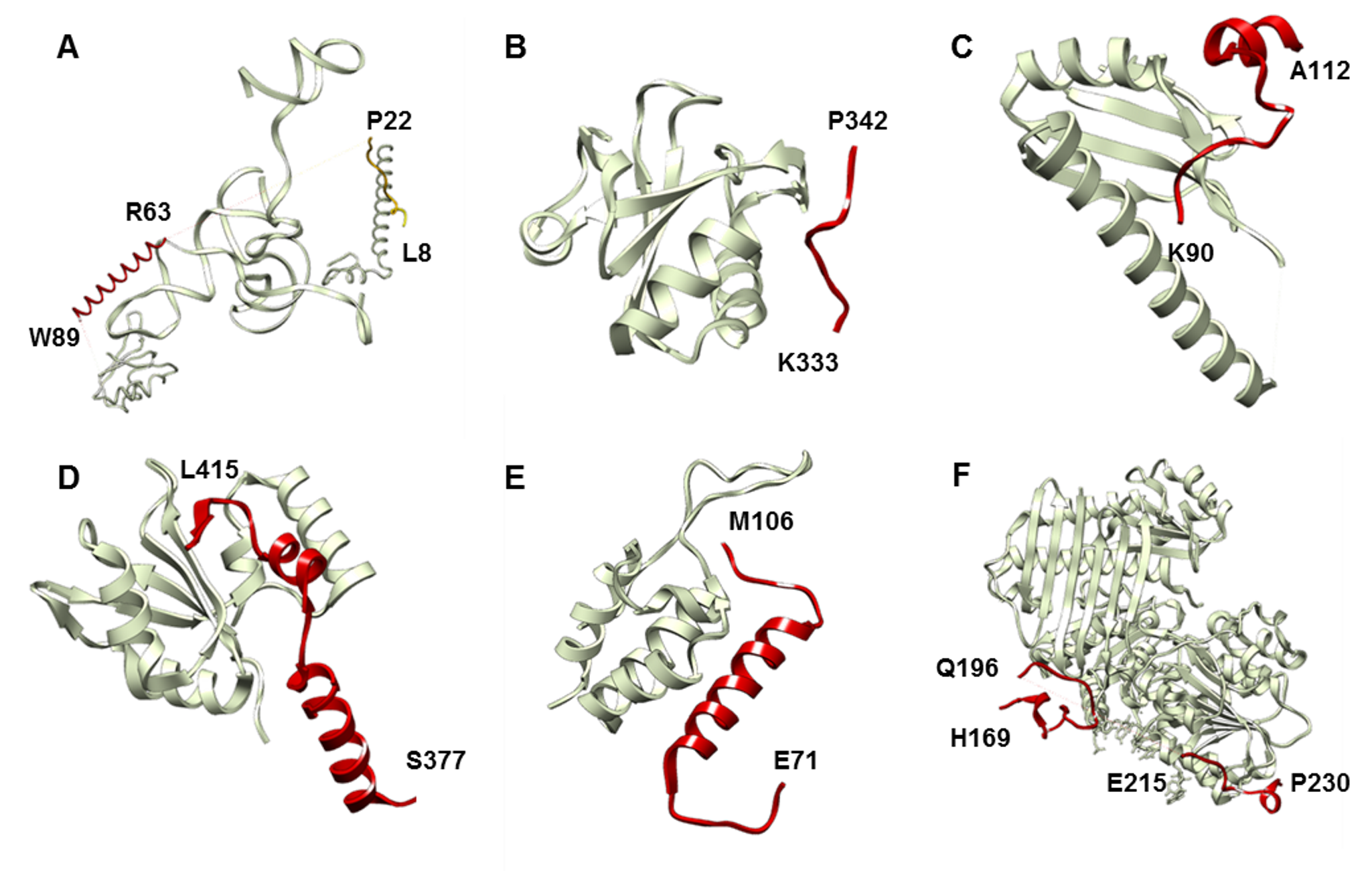

Supplement: Figure S3 — MoRFs in the structures of spliceosome proteins. A: N-U1snRNP70_N (in yellow) and C-U1snRNP70_N (in red) (protein U1-70K in the structure of U1 snRNP with removed Sm proteins, PDB ID: 3CW1). B: ULM (protein SF3b155 in complex with SPF45, PDB ID: 2PEH). C: ULM (protein U2AF65 in complex with U2AF35, PDB ID: 1JMT). D: SF3b1 (protein SF3b155 in complex with SF3b14a/p14, PDB ID: 2F9D). E: SF3a60_bindingd (protein SF3a60 in complex with SF3a120, PDB ID: 2DT7). F: Btz (protein MLN51 in the structure of the exon-junction complex, PDB ID: 2J0S). (TIF) [file pcbi.1002641.s003.tif]

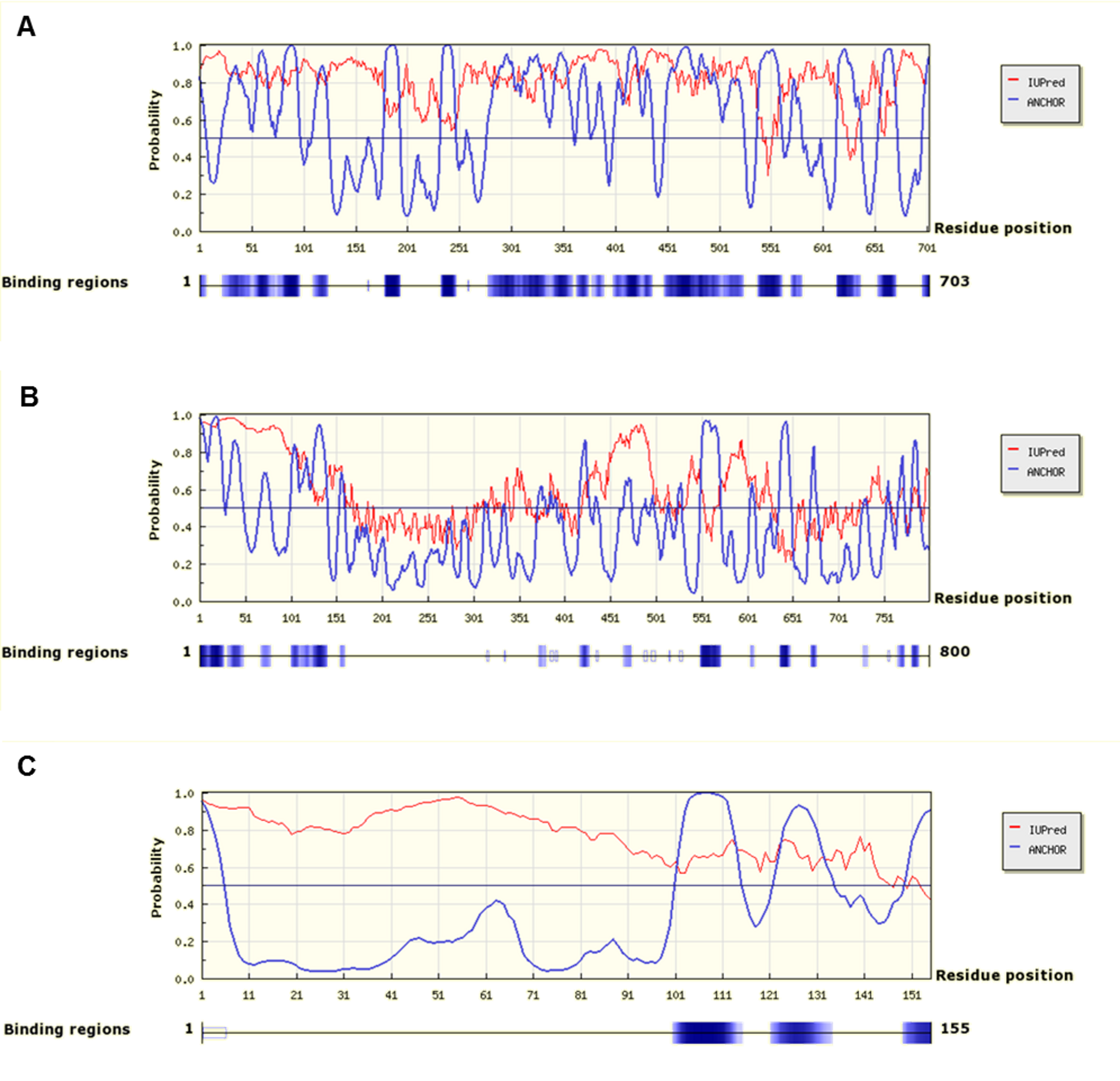

Supplement: Figure S4 — Disorder plots for highly disordered spliceosome proteins. Example disorder plots created by the ANCHOR server, http://anchor.enzim.hu. Red line: disorder probability; blue line: probability of binding another molecule at the residue; blue line at the bottom: another representation of the binding probability (the darker the blue, the higher the probability). A. MLN51 (EJC protein). The region corresponding to the Btz MoRF lies between residues 169–230. B. U4/U6.U5-110K. C. U4/U6.U5-27K. (TIF) [file pcbi.1002641.s004.tif]

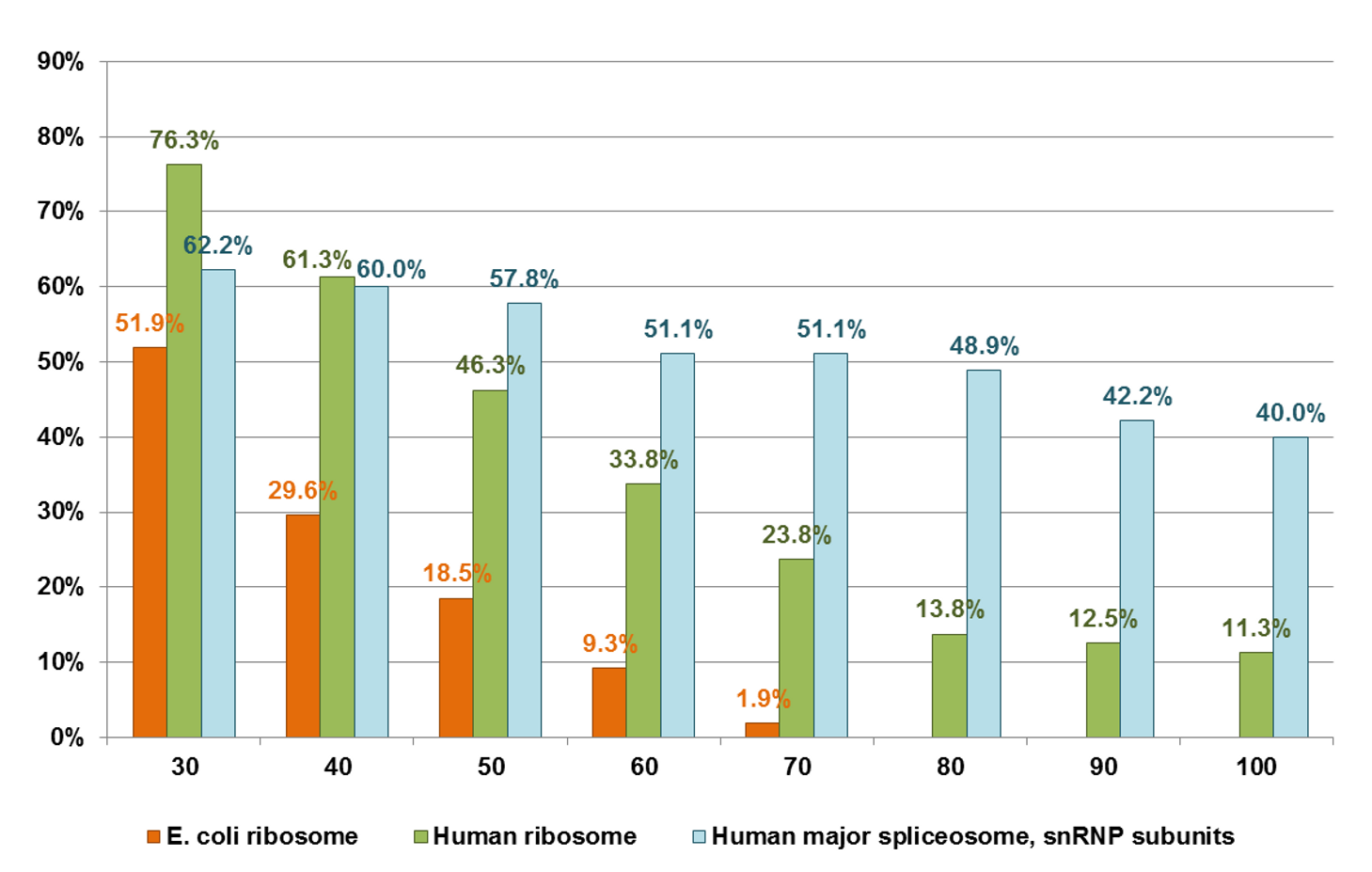

Supplement: Figure S5 — IDR lengths in E. coli and human ribosome and human major spliceosome snRNP subunits. This graph shows the fraction of proteins in the proteomes of the E. coli (orange) and human ribosome (green) and the snRNP subunits of the major spliceosome (blue) that contain at least one IDR of a given length. (TIF) [file pcbi.1002641.s005.tif]

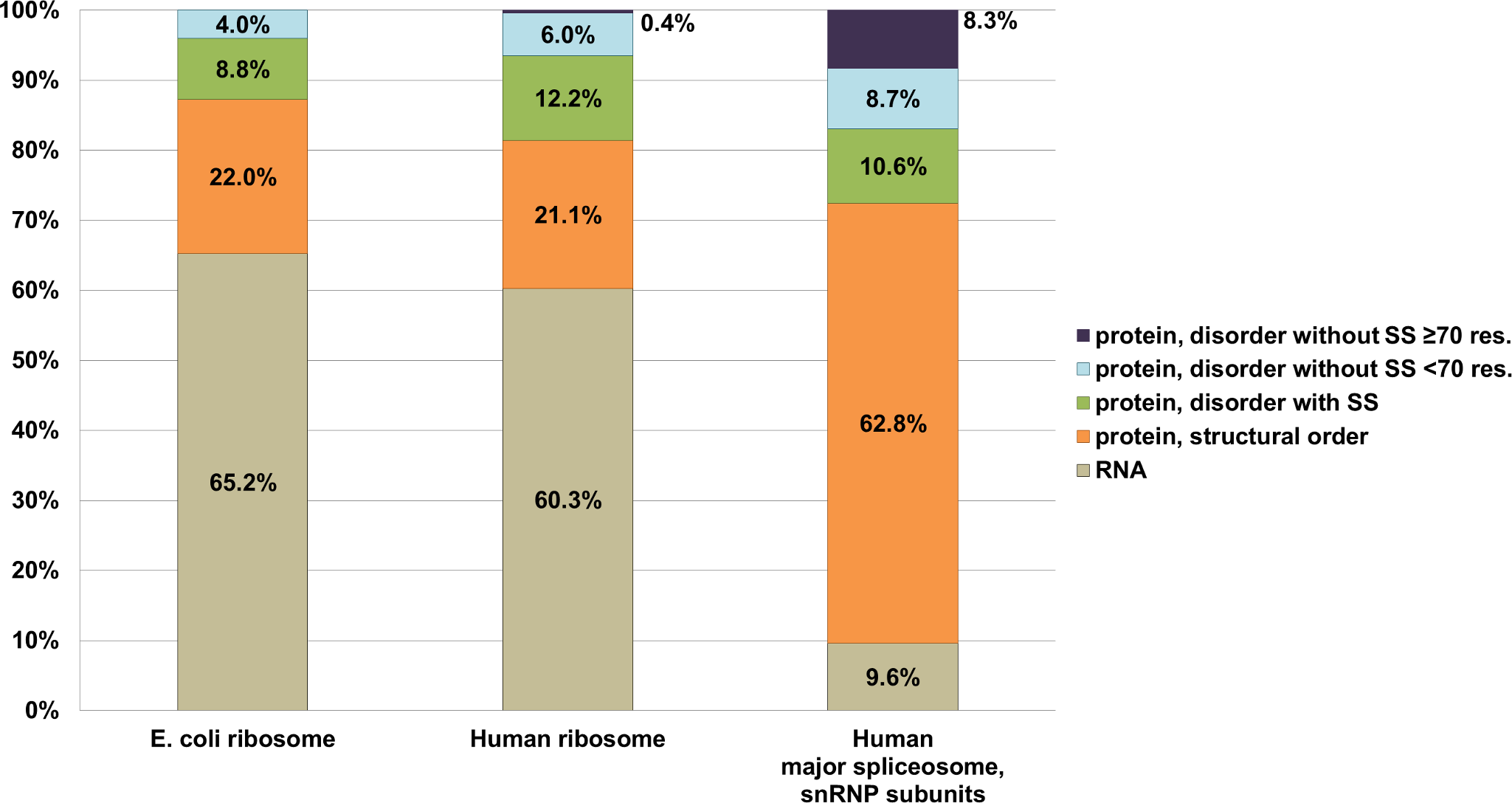

Supplement: Figure S6 — Structural regions in E. coli and human ribosome and human major spliceosome snRNP subunits. This graphs shows the fractions of the total weight of the three complexes taken up by different types of structural regions. The Sm proteins were calculated four times each towards the weight of the spliceosome. (TIF) [file pcbi.1002641.s006.tif]

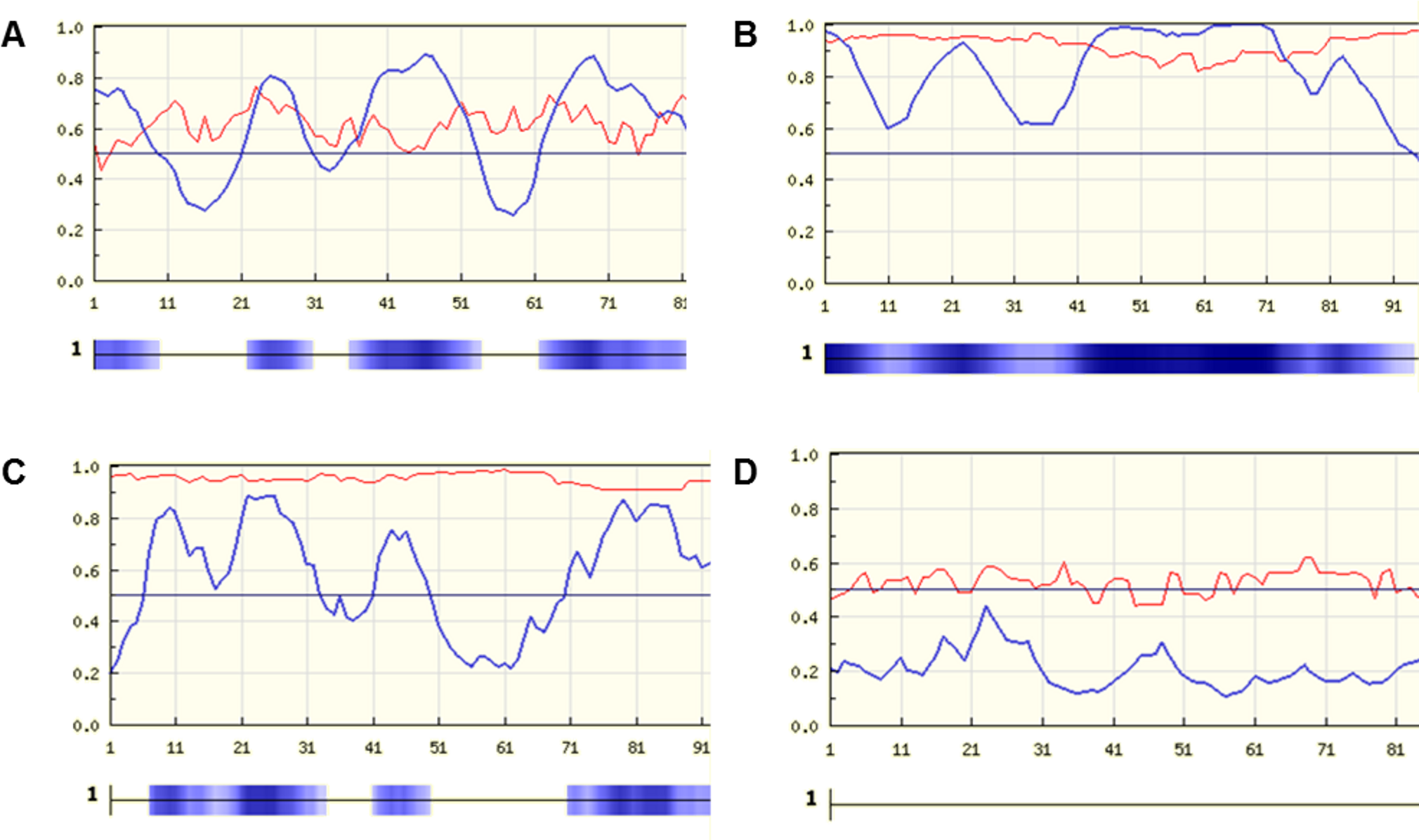

Supplement: Figure S7 — Disorder plots for various types of IDRs found in spliceosome proteins. Example disorder plots created by the ANCHOR server, http://anchor.enzim.hu. Red line: disorder probability; blue line: probability of binding another molecule at the residue; blue line at the bottom: another representation of the binding probability (the darker the blue, the higher the probability). A. IDR with SS: SF3b145, residues 738–818; B. RS-like IDR: protein 9G8, residues 121–215; C. polyP/Q IDR: SF3a66, residues 216–307; D. hnRNP G-rich IDR: hnRNPA1, residues 200–285. Interpretation of the plots: A is predicted to contain short regions of order in regions of disorder, B and C are predicted to be almost completely unfolded in isolation and D is largely insoluble. A, B and C contain regions predicted to be binding. In the case of the RS region, this encompassed almost its entire length. (TIF) [file pcbi.1002641.s007.tif]
